# Adipose-Derived Mesenchymal Stem Cells: A Promising Tool in the Treatment of Musculoskeletal Diseases

**DOI:** 10.3390/ijms20123105

**Published:** 2019-06-25

**Authors:** Marta Torres-Torrillas, Monica Rubio, Elena Damia, Belen Cuervo, Ayla del Romero, Pau Peláez, Deborah Chicharro, Laura Miguel, Joaquin J. Sopena

**Affiliations:** 1 Bioregenerative Medicine and Applied Surgery Research Group, Department of Animal Medicine and Surgery, CEU Cardenal Herrera University, CEU Universities, C/Tirant lo Blanc, 7, Alfara del Patriarca, 46115 Valencia, Spain; marta.torrestorrillas@uchceu.es (M.T.-T.); elena.damia@uchceu.es (E.D.); belen.cuervo@uchceu.es (B.C.); ayla.delromero@uchceu.es (A.d.R.); pau.pelaez@uchceu.es (P.P.); debora.chicharro@uchceu.es (D.C.); laura.miguel@uchceu.es (L.M.); jsopena@uchceu.es (J.J.S.); 2 García Cugat Foundation CEU-UCH Chair of Medicine and Regenerative Surgery, CEU Cardenal Herrera University, CEU Universities, C/Tirant lo Blanc, 7, Alfara del Patriarca, 46115 Valencia, Spain

**Keywords:** adipose-derived mesenchymal stem cells, musculoskeletal diseases, regenerative medicine

## Abstract

Chronic musculoskeletal (MSK) pain is one of the most common medical complaints worldwide and musculoskeletal injuries have an enormous social and economical impact. Current pharmacological and surgical treatments aim to relief pain and restore function; however, unsatiscactory outcomes are commonly reported. In order to find an accurate treatment to such pathologies, over the last years, there has been a significantly increasing interest in cellular therapies, such as adipose-derived mesenchymal stem cells (AMSCs). These cells represent a relatively new strategy in regenerative medicine, with many potential applications, especially regarding MSK disorders, and preclinical and clinical studies have demonstrated their efficacy in muscle, tendon, bone and cartilage regeneration. Nevertheless, several worries about their safety and side effects at long-term remain unsolved. This article aims to review the current state of AMSCs therapy in the treatment of several MSK diseases and their clinical applications in veterinary and human medicine.

## 1. Introduction

Given the increase in life expectancy, and the global trend to obesity, degenerative changes, including loss of bone density, ligament and tendon flexibility, and osteoarthritic changes, together with traumatic injuries, are becoming an enormous medical and socio-economical burden. These musculoskeletal (MSK) disorders cause both pain and functional defects [1,2,3]. The Bone and Joint Decade initiative, led by the United Nations and the World Health Organization, reported that chronic MSK pain is the most common medical complaint in the United States [3].

Due to the tremendous impact of MSK injuries, efforts are being made in order to find appropriate cell-based and tissue engineering therapies for bones, tendons, joints, and skeletal muscle disorders [1]. Of particular interest are mesenchymal stem cells (MSCs).

At this time, two different descriptions of MSCs are found in the literature. Initially, MSCs were described as stem cells only located in the bone marrow with the capacity to regenerate bone and to create the niche for hematopoietic stem cells, now commonly known as bone-marrow mesenchymal stem cells (BM-MSCs) [4]. The other view sees MSCs as ubiquitous multipotent adult stem cells, which can be isolated from many tissues including adipose tissue, muscles, tendons, synovial capsule, dental pulp, skin, lungs, placenta, and umbilical chord [5,6]. MSCs exhibit great differentiation potential into many different types of tissues, including bone, cartilage, muscle, and tendons [7,8,9]; however, stem cells obtained from different tissues can show diverse differentiation capacities, and thus, the term MSCs could be confusing [10].

Three mechanisms for MSCs- based therapy have been proposed: firstly, MSCs can differentiate into the targeted cell type and contribute to repair the damaged area; secondly, they have a paracrine effect, being capable of secret various cytokines and growth factors (GF) to adjacent cells, which leads to vascularization and cellular proliferation in wounded tissues; and thirdly, MSCs have immunomodulatory properties, so they can reduce inflammation in injured tissues [11,12,13,14,15]. In addition, there is no ethical issue for the use of MSCs [16].

MSCs most commonly used are BM-MSCs and adipose-derived mesenchymal stem cells (AMSCs). They share similar properties, but AMSCs offer some advantages compared to BM-MSCs. AMSCs are more accessible and easier to collect from subcutaneous tissue, they can be collected in large quantities, with less morbidity of the patients, via lipoaspirates or adipose tissue biposy, they are easily isolated, and can simply be expanded in vitro [17,18,19,20]. Moreover, AMSCs have been shown to be immunoprivileged, with low risk of rejection, and more genetically stable in long term culture, with a greater proliferative rate than BM-MSCs [21,22,23,24].

Subcutaneous fat tissue is the most accessible source to obtain AMSCs, but in the last years, Tangchitphisut et al., Muñoz-Criado et al., and Spasovski et al. have demonstrated that supra- and infra-patellar fat pads are suitable sources to obtain AMSCs [25,26,27].

After an enzymatic digestion, adipose tissue yields a heterogeneous population of many cell types, which upon isolation are termed the stromal vascular fraction (SVF) [21]. The SVF contain a mixture of regenerative cell types and various immune cells, including preadypocytes, fibroblasts, vascular smooth muscle cells, endothelial cells, resident monocyte, and lymphocytes, but mature adipocytes are absent [28,29,30]. The SVF includes AMSCs, which account for 30% of the total SVF cells [21]. Some studies have shown improved results with the application of SVF compared to AMSCs, promoting cartilage and subchondral bone regeneration [31], forming new cartilage matrix [32], and enhancing the proliferation of myoblasts [30]. In contrast, SVF inhibits the differentiation of myoblasts [30].

AMSCs were first identified in the early 2000s [33]. They are adult stem cells that originate in mesodermal embryonic layer, and they are characterized by their adherence to plastic in culture, their immunophenotype in undifferentiated state, and their self-renewal and multipotency properties [34]. Due to these last two characteristics, AMSCs are able to proliferate to maintain stem cells reservoir in the undifferentiated state during life [35,36,37,38], and give rise to different types of mesenchymal cell lineages (adipocytes, fibrocytes, osteocytes, and chondrocytes) [36,38,39,40,41,42], as well as non-mesenchymal cell lineages (skeletal, smooth and cardiac myocytes, hepatocytes, and neuronal) [39,43,44,45,46,47].

In addition, AMSCs induce a trophic effect through the secretion of a large number of GF and anti-inflammatory proteins in response to inflammatory molecules, including prostaglandin 2, hepatocyte growth factor (HGF), transforming growth factor-β1 (TGF-β1), vascular endothelial growth factor (VEGF), tumor necrosis factor-α (TNF-α), stromal cell-derived factor-1 (SDF-1), nitrous oxide, IL (interleukin)-4, IL-6, IL-10, and IL-1 receptor antagonist, that support the angiogenesis, tissue remodeling, and antiapoptotic events [48,49,50]. Moreover, AMSCs prevent proliferation and function of many inflammatory immune cells, such as natural killer cells, B cells, T cells, monocytes, macrophages, and dendritic cells [51].

Regenerative medicine (RM) is gaining interest among orthopedics clinicians and surgeons due to the showed therapeutic abilities, the increasing prevalence of MSK diseases, and the lack of conventional treatments with good long-term outcomes. During the last years, the clinical potential of AMSCs in the treatment of many MSK diseases has been demonstrated in several studies. Consequently, the aim of the present study is to review the application of AMSCs in the treatment of different MSK pathologies. With this purpose, MSK diseases will be classified in four groups: (i) muscle, (ii) tendon and ligament, (iii) bone, and (iv) cartilage.

## 2. AMSCs in the Treatment of Muscular Disorders

Although muscle has some inherent regeneration capacity to repair damage resulting from physical or chemical trauma to muscle tissue, complete functional recovery of skeletal muscle after severe injury remains a challenge [52,53]. The return of muscular function is comprised by the incomplete recovery from muscle atrophy and fibrosis, which affects muscle fiber number, muscle cross-sectional area, and thus, muscle force [53]. Because of the lack of therapeutic options that decrease fibrosis and delay muscle atrophy, significant efforts are being made to improve the current treatment of skeletal muscle injury using a RM approach. Even so, there is a scarcity of literature regarding the use of AMSCs in skeletal muscle’s injuries.

Previous works detailing the myogenic potential of AMSCs have been performed with mouse and human AMSCs [54]. Zuk et al. were the first to describe in vitro muscle differentiation in AMSCs isolates from humans [55]. While some in vitro works have shown direct formation of myotubes from AMSCs cultures [55,56,57], most investigations have shown that in vitro myogenic differentiation of AMSCs requires co-culture with myoblasts or satellite cells [52,54,58,59,60,61,62,63]. Even though the in vitro differentiation potential of AMSCs into myocytes has been demonstrated, there is little evidence about AMSCs transplantation after skeletal muscle injury. Bacou et al. were the first to test the potential of AMSCs in a nonphysiological cardiotoxin induced muscle damage model in rabbits. Fifteen days after the transplantation, the cells expressed skeletal muscle markers, suggesting myogenic differentiation. In addition, two months after the treatment, muscles were heavier, showed a significantly larger fiber section area, and developed a significantly higher maximal force compared with damaged control muscles [43].

More recent studies, suggested that AMSCs promote the proliferation of myoblasts, which could explain the regenerative capacity shown in vivo [30,63,64,65,66]. Based in these results, during the last years, some animal models have shown the influence of AMSCs in the treatment of some miopathies [67,68,69,70] and muscular dystrophy [63,65,66]. Even so, there is a shortage of literature regarding the application of AMSCs in muscular injuries.

Muscular tears or lacerations are common lesions within athletes. Conventional therapies include medical management with non-steroideal anti-inflammatory drugs (NSAIDs), antioxidant therapy, and steroids; or surgical management such as myotenectomy or myectomy [67,69]. These therapies are unsuccessful and generally do not prevent the formation of fibrous tissue as well as do not promote muscle regeneration, leading to a limited function of the limb [67,68]. Therefore, regenerative therapies are gaining interest, particularly AMSCs, and some animal studies have been carried out in order to assess the in vivo efficacy of AMSCs in muscular tears. Peçanha et al. investigated how AMSCs contribute to skeletal muscle healing after a surgically performed laceration in the rat model. They conclude that AMSCs may accelerate the process of muscle repair, since the number of regenerating muscle fibers and muscle developed force significantly increased in the treated group [69]. Recently, Gorecka et al. have demonstrated that AMSCs transplantation into acute damaged skeletal muscle in a mice model, significantly improves functional muscle tissue regeneration without direct participation in muscle fiber formation [70].

Based in these results, Brown et al. and Gibson et al. carried out clinical studies in which they applied AMSCs in dogs suffering from semitendinosus tears, a pathology with a high incidence in working dogs. The dogs were clinically and ultrasonographic evaluated before and after the local injection of AMSCs. After the AMSCs treatment, all cases in the studies showed improvement in gait analysis and mobility range in short- and long-term follow up. Furthermore, sonographic examination revealed significant reduction in lesion size, and an increase of organized and orientated fibers [67,68] (Table 1). Despite these favorable results, further in vivo studies and controlled clinical trials are necessary for the complete evaluation of the therapeutic benefits of AMSCs therapy in muscular tears or lacerations.

RM is also gaining interest in the field of muscular dystrophies. Duchenne muscular dystrophy (DMD) is the most common and most severe form of muscular dystrophy. It is an X-linked genetic disorder caused by mutations in the dystrophin gene, which cause dystrophin deficiency. The loss of dystrophin leads to a breakdown of the structural integrity of myofibers, resulting in progressive myofibers necrosis, fibroblast proliferation, and growth of fibrous tissue and fat [71]. DMD is a progressive and lethal degenerating disease that affects both skeletal and cardiac muscle. Thus far, there is no effective treatment for DMD; however, several studies on cell therapy, including the application of MSCs have became a promising treatment to restore dystrophin in DMD patients. The use of AMSCs has also been proposed, but this therapy is still in preliminary testing and more experiments are required [53,63,65,66]. Animal models have become increasingly important for testing these regenerative therapies. Mdx mouse is the most widely used animal model for DMD, presenting the same molecular and protein defect as seen in humans with the disease [72].

Pinheiro et al. investigated the effects of AMSCs transplantation on degeneration, regeneration, and skeletal muscle function in Mdx mice. It was firstly observed that AMSCs treatment increases the content of VEGF and anti-inflammatory cytokines in dystrophin-deficient skeletal muscle, which promotes angiogenesis and reduces the inflammation, respectively. Moreover, a decrease in the content of TNF-α and Interleukin 6 (IL-6) were also reported in the AMSCs treated group, suggesting a protective action of the AMSCs on inflammation-induced injury. The AMSCs treated group showed improved muscle strength and resistance to acute muscle fatigue, after one injection of AMSCs per week during four weeks. In addition, a histological analysis was performed and an increase in fiber cross-sectional area and an augment of myogenin content was observed in AMSCs treated group [65]. Similar results were obtained by Lee et al., who demonstrated that AMSCs up-regulated myogenin, mTOR and raptor proteins, which contribute to the formation of myofibres. Thus, leads to an increase in muscle size when compared to control group [66]. These results support the proposition that AMSCs transplantation is a promising treatment for muscular dystrophies. Nevertheless, there is no clinical trials supporting these results; hence, further investigation is needed to determine the long-term effects of AMSCs in dystrophin-deficient muscles, and to find an accurate therapy for this pathology.

## 3. AMSCs in the Treatment of Tendon Injuries

The high incidence of tendon injuries is mainly associated with sport practice and aging, and they range from acute traumatic ruptures to chronic tendinopathy [73,74]. Tendon injuries represent a clinical challenge because their natural repair process is slow, complex, and inefficient, as well as a financial challenge. Tendon has limited inherent healing capacity, as it is a slightly cellular and poorly vascularized tissue, and often responds inadequately to treatments; hence, prolonged recovery times are needed [73,74,75]. After injuries, the structural composition and organization of tendons, which are responsible of the specific mechanical tendon properties, are not completely restored. Following the repair process, a fibrous scar is formed, causing significant dysfunction and joint movement inability, leading to a biomechanically weakened tendon, making it more vulnerable to re-rupture [74,76]. Tendinopathies and tendon tears have been treated with conservative approaches for managing symptoms, including rest, anti-inflammatory drugs, corticosteroids, and physiotherapy. On the other hand, the gold standard to treat tendon rupture is surgical suture, combined or not with allo- or auto-grafts [73,77]. Despite the improvements made in surgical techniques, none of the therapeutic options have provided successful long-term solutions [74,75]. New treatments are needed with the objective of improving tendon regeneration, and AMSCs have been adopted to repair tendon and ligament tears. It has been demonstrated that AMSCs can differentiate in vitro towards tenocytes. It is widely known that the control of stem cell activity is influenced by several environmental factors, including GF such as insulin like growth factor (IGF), TGF-β and growth differentiation factor 5 (GDF-5), which have been successfully used in a co-culture wit primary tenocytes, to promote AMSCs differentiation towards tenocytes in vitro [74,78,79].

During the last decade, some experimental studies have shown the efficiency of AMSCs in animal models of tendon injury. A study carried out by Uysal and Mizuno in mice showed that local administration of AMSCs accelerates tendon repair, as exhibited by an increase in tensile strength, direct differentiation of AMSCs into tenocytes, and increases in angiogenic GFs [80]. Similar results were obtained by the same research group in a rabbit calcaneal tendon injury model, which showed that the application of AMSCs associated with platelet rich plasma (PRP) increases the resistance of tendons as well as the amount of collagen type I, VEGF, and FGF [81]. Vieira et al. were the first investigation team that treated ruptured Achilles tendon of rabbits with AMSCs alone, with no surgical suture. The histological analysis showed a significant increase in capillaries and in the structural organization of collagen in the AMSCs treated group, compared with the surgically sutured group [82]. A rat supaspinatus tendon injury model was conducted by Valencia et al. Histological examination showed less acute inflammation in AMSCs treated group, but controversially, there were no differences in the orientation of collagen fibers between groups. In addition, and contrary to previous studies, none biomechanical differences between the AMSCs treated group and the untreated group were reported [83].

More recent animal models showed comparable results. Oshita et al. conducted a collagenase induced tendinopathy in a rat model and the findings demonstrated that the application of AMSCs results in significant improvement in the pathological findings associated with tendinopoathy [84]. Results reported from a rat tendon injury model demonstrated that tendons treated with AMSCs showed better gross morphological and biomechanical recovery that those in fibrin and control group [85]. Aparecida de Aro et al. demonstrated that AMSCs combined with GDF-5 increases the organization of collagen fibers in the injury adjacent region, which was reflected in biomechanical properties, in a transected Achilles tendon model in mice. Compared with untreated tendons, tendons treated with AMSCs were more resistant to traction, with lower deformation at higher stress [74]. Similar results were obtained in a rotator cuff tear model in rabbits [86]. The results demonstrated that the maximum load, the maximum strength, and stiffness were significantly increased in the group treated with adipose SVF, compared with those of the control group. Furthermore, the results showed that adipose SVF accelerate the transformation of collagen fibers type III into type I [86].

AMSCs are also gaining interest among veterinarians. Race horses often suffer from superficial flexor digitorium longus tendon (SFDLT) lesions. There is clinical evidence that the injection of AMSCs after SFDLT spontaneous lesion significantly improves healing [87]. AMSCs were administered under ultrasonographic guidance in four horses suffering from SFDLT lesions. Treated horses showed shorter periods of lameness and better organization of collagen fibers assessed by ultrasound examination [87]. These results concur with those obtained by Carvalho et al., in a horse’s collagenase-induced SFDLT lesion controlled trial [88]. The histological evaluation demonstrated that AMSCs combined with a platelet concentrate therapy resulted in a better organization of collagen fibers and a decrease of the inflammatory infiltrate. In addition, the ultrasound evaluation showed a lack of lesion progression in the treated group [88] (Table 2).

Even though experimental animal models and clinical trials have shown benefits of AMSCs therapy in tendon injuries at biomechanical, histological, and molecular levels, the clinical use of AMSCs in the treatment of tendinopathy has not been well-studied.

The first case report using AMSCs to treat chronic tendinopathy was conducted by Lee et al. Twelve patients with lateral epicondylosis, a degenerative condition in the wrist extensor tendons, were administered with AMSCs under ultrasound guidance, and clinical and ultrasound evaluation were performed at 6, 12, 26, and 52 weeks after treatment. Results demonstrated that AMSCs therapy was effective in improving elbow pain (evaluated by visual analogue scale (VAS) score), performance (evaluated by modified Mayo clinic performance index), and structural defects (evaluated with ultrasound images of tendon defect area) [77].

Kim et al. studied the effectiveness of an injection of AMSCs loaded in fibrin glue during arthroscopic rotator cuff repair. 70 patients were divided into two groups; one group was only treated with the arthroscopy, and the other group was treated with the arthroscopy combined with single injection of AMSCs. Clinical (VAS score and range of movement (ROM)) and magnetic resonance imaging (MRI) outcomes were assessed. MRI results at a minimum of 12 months after surgery revealed that the rotator cuff tendon was completely healed in 71% of patients in the conventional group, and in 83% of patients in the AMSCs group. In addition, the retear rate was significantly lower in AMSCs group (14.3%), compared with conventional group (28.6%). On the contrary, no differences between groups were observed in VAS scale and ROM [89].

The efficacy of SVF injection in the treatment of Achilles tendinopathy has also been studied by Usuelli et al. [90]. The clinical controlled trial aimed to compare the effectiveness of the injection of PRP, with the injection of adipose-derived SVF for the treatment of chronic Achilles tendinopathy. Fifty-six patients affected by non-insertional Achilles tendinopathy were randomly divided into PRP or SVF treatment group. Either PRP or SVF was locally injected and a clinical exam was carried out at 15, 30, 60, 120, and 180 days after treatment. Moreover, ultrasound and MRI examination were conducted 4 and 6 months after treatment. Results showed that both, PRP and SVF provide a significant clinical improvement in terms of pain relief and function restoration. Interestingly, a faster recovery was reported in the SVF group. Despite the positive clinical outcome, none treatment group showed ultrasound or MRI improvements [90].

Considering the above-mentioned points, one can conclude that AMSCs transplantation is a good alternative for the treatment or tendinopathies and tendon ruptures, but further clinical trials and case reports are needed.

## 4. Application of AMSCs in the Treatment of Osseous Diseases

AMSCs can differentiate into different cell types, including osteocytes, and the lineage-specific differentiation is associated with the expression of explicit phenotypic markers and mature tissue genes. In vitro, osteogenic differentiation of AMSCs can be obtained using medium supplemented with ascorbic acid, b-glyc-erophosphate, dexamethasone, 1.25 vitamin D_3_ or bone morphogenic protein 2 [91,92,93,94,95]. Osteogenic induction was thought to be a necessary step for AMSCs to have osteogenic ability, but it has been demonstrated that AMSCs undergoing, or not, osteogenic induction are able to adhere to scaffolds, migrate, proliferate, and differentiate when transplanted in bone tissue in vivo [96,97].

Bone fractures, segmental bone defects and critical size defects (CSDs) are important causes of patient morbidity and place an incredible economic burden on the healthcare system. They are usually secondary to trauma, post-resection of tumors, or post-debridement of infection [17]. Around 5 billion dollars are annually spend in treating bone defects in the US, mainly on bone grafts and implants for bone injuries and other pathologies associated with defective fracture healing, such as non-union [98]. Conventional treatments, including autologous bone grafts, and distraction osteogenesis (DO) have some limitations, such as long immobilization periods, donor site morbidity, muscular atrophy and surgical complications such as infection, pain, or hemorrhage [17,99,100]. Tissue engineering and cell-based therapies have been adopted as alternatives therapies to promote bone repair, and AMSCs have been proposed to treat CSDs [100,101,102,103,104,105,106] and delayed fracture healing and the resulting segmental bone defects [99,107,108,109,110]. Furthermore, the implication of AMSCs in DO in animal models has been investigated [111,112].

Levi et al. introduced PLGA scaffolds alone or PLGA scaffolds with AMSCs in critical size calvarial defects in mice, and near complete healing was observed among AMSCs engrafted calvarial defects in comparison to control group, that showed little healing [102]. Liu et al. [100], who previously demonstrated that autologous AMSCs loaded onto natural coral scaffolds could repair cranial CSDs in a canine model [106], were the first to show that allogenic AMSCs combined with coral scaffolds are suitable to regenerate the same kind of defects without using immunosuppressive therapy [100]. Critical tibial defects treated with hydroxyapatide scaffolds combined with AMSCs showed an improved healing process when compared to that occurred when only the scaffold was used [105]. Moreover, the defects treated with AMSCs showed greater mechanical properties, suggesting an enhanced ability to bear mechanical loading [105]. Du et al. combined the osteogenesis and angiogenesis advantages of AMSCs with modified mesoporous bioactive glass scaffolds to optimize the restoration of CSDs, and the results demonstrated that the combination of different induction of AMSCs into osteogenic cells and endothelial cells is practical and beneficial for CSDs [104]. To our knowledge, there is only a case report regarding the use of AMSCs in the treatment of CSDs. Lendeckel et al. described the use of AMSCs combined with bone graft and fibrin glue to treat cranial CSD in a seven-year-old girl suffering from multiple calvarial fractures. Three-month follow-up computed tomography scan revealed new bone formation and almost complete calvarial continuity [113].

Studies regarding the use of AMSCs during DO are scarce in the literature. DO has been a very successful technique that is being used worldwide to treat multiple orthopedic conditions; however, the fixator needs to be kept in place a long time until consolidation is done [17]. The therapeutic potential of AMSCs in tibial defects managed by DO was investigated in a rabbit model by Sunay et al., and they concluded that osteoblasts-differentiated AMSCs shorten the consolidation period of DO [112]. Radiologic analyses of rabbits treated with osteoblast-differentiated AMSCs revealed increased callus density and ossification rate compared with rabbits treated with undifferentiated AMSCs. In addition, biomechanical tests showed that the highest ossification rate was observed in osteoblast-differentiated AMSCs group, and histopathologic studies showed that the quality of newly formed bone was significantly higher in the same group [112]. Nomura et al. investigated whether uncultured-AMSCs combined with collagen gel could promote bone formation in rats DO model [111]. The results demonstrated that bone density of the distracted callus in the AMSCs group was significantly increased in comparison with control group at 6 weeks after injection, and the fracture strength in AMSCs group was significantly higher. In addition, real-time reverse transcription-polymerase chain reaction of the AMSCs formed callus showed both osteogenic differentiation and secretion of growth factors [111].

Concerning the application of AMSCs in the treatment of bone fractures, in a case report by Saxer et al., autologous adipose SVF was loaded onto ceramic granules within fibrin gel and used to treat humeral fractures in eight patients along with standard open reduction and internal fixation [109]. Biopsies of the repair tissue 12 months after the transplantation of AMSCs, demonstrated formation of bone ossicles, structurally disconnected and morphologicaly distinct from osteoconducted bone, suggesting the osteogenic and angiogenic nature of implanted SVF cells [109]. Anti-inflammatory effects of AMSCs have also been demonstrated in an equine bone fracture case report [110]. Lee et al. analyzed synovial fluid of racehorses suffering bone fractures before and after the intra-articular (IA) injection of AMSCs, and the level of pro-inflammatory factors was significantly decreased in synovial fluids of AMSCs treated horses [110].

Despite advances in fractures treatments, annually, over 5–10% of bone fractures are not able to union [99]. In a study carried out by Ghasroldasht et al., a combination of AMSCs, cancellous bone graft and chitostan hydrogel was applied to non-union bone fractures in rats [99]. As early as 2 weeks after surgery, radiological assessment revealed that callus formation started at the fracture site and higher healing grade was observed in the AMSCs group. Histological and biomechanical studies 8 weeks after surgery supported these findings. It was observed that the defect was filled with hard callus in AMSCs group, and additionally, higher stiffness and ultimate load were reported in the treated group compared to control [99]. On the contrary, Dozza et al. concluded that AMSCs do not improve the healing process when cultivated on demineralized bone matrix before implantation, in a sheep nonunion fracture model [108].

Age-related skeletal changes, such as osteoporosis, are closely related to imbalanced bone remodeling characterized by elevated osteocyte apoptosis and osteoclast activation [114]. Recently, a study has demonstrated for the first time that AMSCs exosomes inhibits induced osteocyte apoptosis and osteocyte-mediated osteoclastogenesis [115]. The use of AMSCs as a regenerative therapy for osteoporosis is a topic of current interest, as it could potentially reduce the susceptibility of fractures and increase lost mineral density [116]. Mirsaidi et al. evaluated the use of AMSCs as a treatment strategy for age-related osteoporosis, both in vitro and in vivo [117]. The research group demonstrated that AMSCs isolated from osteoporotic mice have the ability to undergo osteogenic differentiation and induce mineralized tissue formation. In addition, a single intratibial injection of AMSCs significantly improved trabecular bone quality after 6 weeks in comparison to untreated contralateral bones [117]. Similar results were obtained by Uri et al. in a study in which AMSCs- seeded scaffolds were implanted into the proximal femur of osteoporotic rats [114]. Results showed that the mean cortical thickness, bone volume density, and bone load to failure in AMSCs injected femora were higher compared to control group. Furthermore, completed osseo-integration of the scaffolds and no inflammatory response were reported [114]. Future clinical studies are still needed to validate that human AMSCs have the ability to regenerate lost bone mass in osteoporotic patients.

Avascular necrosis (AVN), also known as osteonecrosis, of the femoral head, is a debilitating disorder that causes necrosis, bone structure collapses, bone destruction, and consequently, pain and joint dysfunction [118]. Despite that BM-MSCs have been used as a cellular therapeutic option for treatment of AVN of the femoral head, limited success has been achieved. Wyles et al. demonstrated that AMSCs outperformed BM-MSCs in growth rate and bone differentiation potential in the setting of AVN, suggesting they could provide a more-potent regenerative therapeutic strategy [119]. In 2011, a series of clinical case reports demonstrated that AMSCs injection, in conjunction with hyaluronic acid (HA) and PRP, is a promising therapy for AVN of femoral head [120]. The MRI data for all patients showed significant positive changes, and subjective pain and functional status improvements [120]. The long-term effect of AMSCs on bone regeneration in patients with AVN has also been reported by Pak [121]. Two patients were involved and they both showed improved symptoms and positive bone regeneration 16 months after treatment [121]. In another case report, Pak et al. treated a man with early stage AVN of the femoral head with a combination of AMSCs, PRP, and HA [118]. The patient was followed-up with MRI scans, VAS, and ROM assessments at 3, 18, and 21 months after treatment. Severe hip pain was considerably improved at 3 months follow up, with ROM and MRI showing near complete resolution. At 18 and 21 months after treatment, complete resolution of AVN was reported [118] (Table 3).

## 5. Application of AMSCs in the Treatment of Cartilage Disorders

The cartilage has poor self-healing ability due to their avascular nature, and hence lack of systemic regulation. After an injury, the repaired tissue is fibrous in nature and does not have the functional properties of native hyaline cartilage, so joint degeneration is accelerated [122]. Recently, MSCs have received increasing attention as promising options for osteochondral regeneration. In vitro studies have demonstrated that AMSCs are able to differentiate towards chondrocytes when they are cultured alone or in combination of GF, such as IGF-1 or TGF-β, and these chondrocytes have the same expression of type II collage that mature chondrocytes [123,124,125]. Rodríguez-Jiménez et al. showed that plasma rich in growth factors (PRGF) positively contributes to viability and proliferation of canine AMSCs into caprolactone 2-(methacryloxy) ethyl estes scaffolds [126].

Bosetti et al. analyzed AMSCs chondroinductive properties in vitro and showed that these cells induce chondrocyte proliferation and extracellular matrix production [127]. Despite the mechanism by which AMSCs cause cartilage regeneration still unclear, it has been postulated that these cells may act on subchondral bone, forming the primary repair cartilage [128]. Moreover, it has been demonstrated that after IA injection of AMSCs, these cells were found in the synovial membrane and they expressed molecules with anti-inflammatory and chondrogenic properties [129]. A recent study has shown that AMSC reduce the secretion of proinflammatory cytokines and protect against apoptosis though autophagy inducing [130]. Some studies have proposed that the use of scaffolds seeded with AMSCs could improve retention, aggregation, viability, proliferation, migration, and chondrogenic differentiation of these cells [131,132,133], while others reported that a scaffold can potentially influence the microenvironment affecting the regional specification of AMSCs [122,134].

Osteoarthritis (OA) is a chronic disease that usually affects older people and damages articular cartilage and synovial joints, leading to pain and disabilities. Almost 80% of elderly population over age 65, and around 12% of global population suffers from OA, and it is considered an economic burden on society, due to the increasing incidence and the high cost of medical treatments [135]. Current pharmaceutical (analgesics, NSAIDs, glucosamine, chondroitin sulfate, and injection of IA therapies such as corticosteroids and HA) or surgical treatments (subchondral drilling and microfracture) aim to decrease pain and improve articular function, but they have limited efficacy in halting OA progression [6]. Over the last few years, regenerative therapies based in the application of stem cells, especially MSCs, and PRP, have emerged as a good therapeutic strategy to treat OA [136]. IA injection of AMSCs is the most commonly reported protocol for AMSCs delivery into the damaged cartilage, however, synovial fluid from arthritic joints is reported to be cytotoxic to cultured AMSCs [137]; thus, new protocols have been developed, such as intravenous administration. It has been demonstrated that AMSCs can exert systemic anti-inflammatory effects following intravenous administration [138].

Several animal models have demonstrated the chondrogenic potential of autologous AMSCs when transplanted into osteochondral defects [31,139,140,141]; however, the use of allogenic AMSCs was first reported by Feng et al. (2019) and results indicated that IA injection of allogenic AMSCs efficiently promoted cartilage regeneration in a sheep model, and these cells survived at least 14 weeks after injection [142]. Similar results were obtained by Oshima et al. (2019) in a rabbit model, in which allogenic AMSCs were able to adhere to the osteochondral defect and promote histological healing [122].

With respect to veterinary medicine, Cuervo et al. demonstrated the safety and effectiveness of a single IA injection AMSCs as a treatment in dogs with hip OA. Functional limitation, ROM, VAS score and patient’s quality of life progressively improved during the 6 months follow-up period. Additionally, better results were obtained in dogs treated with AMSCs than in the ones treated with plasma rich in growth factors [136]. Moreover, Vilar et al. demonstrated that AMSCs therapy improved limb function in dogs suffering from hip OA [143]. A force platform was used before and after the treatment with AMSCs and an increase in peak vertical force (PVF) and vertical impulse (VI) was reported within the first three monts [143]. Recently, Olsen et al. evaluated the safety and the clinical effects of intravenously administered AMSCs in dogs with elbow OA [144]. While some subjective outcome measures, such as activity and behavior of the dogs reported by owners, showed significant improvements, objective outcome measures (synovial fluid biomarkers and mean peak vertical force) did not confirm similar changes [144]; thus, further research is needed before intravenous AMSCs can be recommended as a treatment for OA.

Regarding human medicine, in 2011, for the first time, Pak reported a case series of patients with knee OA treated with adipose SVF. Three months after treatment, the VAS, functional rating index (FRI), and ROM were all improved along with MRI evidence of cartilage regeneration [120]. Hyunchul et al. demonstrated the safety of IA injection of AMSCs for knee OA [145]. Eighteen patients with OA of the knee were randomly assigned to three different groups depending on the AMSCs injected dose (low-dose (1.0 × 10^7^ cells), mid-dose (5.0 × 10^7^), and high dose (1.0 × 10^8^) and had clinical, radiological, arthroscopic and histological evaluations 6 months after injection. After 6 months, the procedure was found to be safe and no treatment-related serious adverse effects were reported. Furthermore, 6 months after treatment, Western Ontario and MCMaster Universities Osteoarthritis Index (WOMAC) score improved and VAS score decreased in the high-dose group; MRI assessment showed that the size of the cartilage defect significantly decreased while the volume of cartilage increased in the high-dose group, whereas no significant changes were observed in the other dose groups. The second-look arthroscopy revealed that regenerated cartilage formed in the most severely degenerated area, and the histology demonstrated thick hyaline-like cartilage regeneration [145]. These same patients were evaluated after a two-year period and no adverse events were reported [146]. The outcome demonstrated that an IA injection of AMSCs improved knee function, as measured with the WOMAC scale, and reduced knee pain, as measured with the VAS, regardless of the cell dosage. In the high-dose group, the size of the cartilage defect measured with MRI decreased from baseline over two years. Clinical and MRI outcomes tended to deteriorate after one year in the low- and medium-dose groups, while those in the high dose group plateaued until two years [146]. Closely similar results were obtained by Pers et al., suggesting that the IA injection of AMSCs is a safe therapeutic alternative to treat severe knee OA patients [147]. Recently, Song et al. carried out a pilot study with three repeated IA injections of AMSCs within 48 h. Eighteen patients were divided into three dose groups (1 × 10^7^, 2 × 10^7^ and 5 × 10^7^). Patients were followed up for 24 months, and results, as seen in previous studies, showed that AMSCs improved pain, function, and cartilage volume of the knee joint, with the high-dose group as the one that exhibited the highest improvement [148]. Spasovski et al. treated nine patients with a single IA injection of AMSCs and they were followed up for 18 months [26]. Significant improvement of clinical scores was observed within first 6 months and persisted during the rest of the follow-up period. MRI visualization showed significant cartilage restoration; on the contrary, radiographic assessment did not show any cartilage improvements [26]. Lately, IA injection of AMSCs combined with arthroscopic debridement has demonstrated to be an effective protocol to improve pain and function in patients with early knee OA [149]. Panni et al. retrospectively analyzed 52 patients with early knee OA that were treated with arthroscopic debridement followed by IA injection of AMSCs, and clinical and functional scores significantly improved in these patients at mid-term follow up (range between 6 to 24 months), especially those with higher pre-operative VAS scores [149].

Several studies have shown that isolated post-traumatic chondral defects and posterior inadequate healing in areas of weight wearing, predispose a patient to later development of OA mainly due to impairment in load transmission. For the management of isolated chondral injuries, surgical interventions, including arthroscopic debridement, microfracture or autologous chondrocytes implantation, are often considered due to the early progression to OA if they are left untreated, but they have some limitations like patient morbidity and inconsistent long-term outcomes [150]. Freitag et al. (2017) reported the successful management of a post-traumatic chondral defect of the patella using IA AMSCs therapy in a patient. After treatment, validated questionnaires for pain and functional assessment (Knee Injury and Osteoarthritis Outcome Score (KOOS), WOMAC, and Numeric Pain Rating Scale (NPRS)) showed consistent improvement across the course of follow-up. Additionally, structural improvements were observed on MRI with complete fill of the chondral defect and smooth integration with the surrounding native cartilage, and MRI T2-mapping techniques indicated normal hyaline-like cartilage regeneration [151]. Similar results were obtained in a patient with a large osteochondral defect of the knee, who suffered from osteochondritis dissecans and with history of multiple unsuccessful past surgical interventions [152]. Following AMSCs therapy, the patient reported improvement in pain and function as measured by NPRS, WOMAC, and KOOS. Moreover, MRI follow-up showed evidence of improvement in cartilage volume and osteochondral architecture at the site of injury, and T2-mapping indicated hyaline-like cartilage morphology [152].

The menisci of human knee are a pair of fibrocartilaginous structures that provide stability and nutrition to articular cartilage and absorb shock to the knee. With knee injuries, the meniscus may be damaged, and once injured, their natural healing potential is very limited. Meniscus tear of the knee is managed conservatively with NSAIDs and physical therapy, but when these treatments fail, arthroscopic meniscectomy is necessary. Such surgery may predispose the joint toward early development of OA [153,154,155]. As an alternative treatment to the surgical repair of meniscal tears, AMSCs have been investigated [154,156,157]. In 2013, Pak et al. reported a cohort study involving 32 patients with degenerative and non-degenerative meniscal tears that were treated with AMSCs combined with PRP and HA. Results showed symptomatic improvements as measured on a VAS and FRI, along with MRI evidence of the meniscal cartilage regeneration [154]. A year later, the same research group reported a case study in which an IA injection of SVF, together with PRP and HA, repaired a meniscal tear in a young patient. Three months after the treatment, the patient’s symptoms significantly improved and almost complete disappearance of the torn meniscus was observed in MRI [156]. Recently, Sasaki et al. have demonstrated that AMSCs-seeded hydrogels enhanced healing of radial meniscal tears in an in vitro model, as histologically evidenced by increased neotissue formation, and healing was further improved by exposure of AMSCs to TGF-β. Additionally, immunohistochemical analysis detected procollagen type II only in the AMSCs-seeded hydrogel groups, and mechanical testing showed higher values of load to failure and stiffness in AMSCs-seeded hydrogels groups [157] (Table 4).

A review of the literature suggests that AMSCs own an intrinsic therapeutic potential that may contribute to cartilage regeneration, thus potentially healing isolated chondral defects, meniscal tears, and OA lesions, offering great potential as an alternative treatment modality.

## 6. Materials and Methods

The authors searched PubMed, Web of Science and Scopus English languages articles using a combination of “adipose derived mesenchymal stem cells”, “tendon”, “ligament”, “cartilage”, “bone”, “muscle”, and “musculoskeletal diseases” as keywords. We chose about 120 articles dedicated to the treatment of different musculoskeletal pathologies with ASCs, and that were published in the last 10 years. Special attention has been drawn to original analysis and studies. Other searches were executed using bibliographies of articles found in the primary and secondary search. One limitation in this review is the fact that our methods, while rigorous, did not follow any formal guidelines for a systematic review (e.g., the Preferred Reporting Items for Systematic Reviews and Meta-Analyses (PRISMA) guidelines).

## 7. Conclusions

The treatment of MSK disorders is gaining interest as the population ages and cell-based tissue engineering has the potential to improve the current treatment of such disorders. During the last years, adipose tissue has emerged as an attractive source of stem cells because their abundance in the body and because AMSCs can be easily isolated using minimally invasive procedures. In addition, they produce larger cell yields than BM-MSCs.

The osteogenic, chondrogenic, and myogenic potential of AMSCs have been widely reported during the last decade. A comprehensive review of the literature suggests that AMSCs posses an inherent therapeutic potential that can directly and indirectly contribute to cartilage, bone, tendon, and muscle regeneration, offering a huge potential as an alternative treatment modality. While there is substantial number of case-reports regarding the clinical use of AMSCs therapies in chondral and bone injuries, the literature with respect to muscle and tendon disorders is scarce.

Although the successful applications of autologous AMSCs may represent a promising therapeutic alternative, many issues, such as their long-term safety, the duration of their effect, the needed AMSCs’ volume to achieve proper outcomes, and the absence of a universal isolation procedure should be resolved and clarified before clinicians can use these treatments on a wider scale. Moreover, the self-renewal and the generation of differentiated progeny of AMSCs in vivo have not been well-documented yet. To this end, animals and humans clinical trials, with longer follow up periods and consistent methodology, are needed.

## Figures and Tables

**Table 1:** Clinical application of AMSCs in muscular injuries

Authors	Patients	Injury	Treatment	AMSCs Origin	Outcomes
Brown et al., 2012 [68]	Dogs	Semitendinosus tear	Locally injected AMSCs	Autologous alciform fat	Reduction in lesion size with well organized fibers. No gait abnormalities.
Gibson et al., 2017 [67]	Dogs	Semitendinosus tear	Locally injected AMSCs	Autologous falciform fat	Reduction in lesion size and VAS. All dogs returned to normal activity within 3 months.

AMSCs: Adipose-derived Mesenchymal stem cells, VAS: Visual Analogue Scale.

**Table 2:** Clinical application of AMSCs in tendon injuries.

Authors	Patients	Injury	Treatment	AMSCs Origin	Outcomes
Lee et al., 2015 [77]	Human	Lateral epicondylosis	Locally injected AMSCs + fibrin glue	Allogenic subcutaneous fat	VAS score improvement. Tendon’s defect size decreased
Skutella, 2016 [87]	Race horses	SFDLT tear	Local injection of AMSCs	Autologous subcutaneous fat	Improvements in gait and lameness assessment. Sonographyc improvement of the defect size and organization of collagen bundles
Kim et al., 2017 [89]	Human	Rotator cuff tear	Arthroscopy + local AMSCs + fibrin glue	Autologous buttock fat pad	Lower retear rate with almost complete healing of the defect by 12 months follow-up
Usuelli et al., 2018 [90]	Human	Non-insertional Achilles tendinopathy	Intratendinous adipose-derived SVF	Autologous abdominal subcutaneous fat	Pain relif and function restoration during at least 6 months

AMSCS: Adipose-derived Mesenchymal Stem Cells, SFDLT: Superficial flexor digitorium longus tendon, SVF: Stromal Vascular Fraction, VAS: Visual Analogue Scale.

**Table 3:** Clinical application of AMSCs in bone injuries.

Authors	Patients	Injury	Treatment	AMSCs Origin	Outcomes
Lendeckel, 2004 [113]	Human	Cranial CSD	AMSCs + fibrin glue + bone graft	Autologous buttock fat	New bone formation and almost comlete calvarial continuity at 3 months
Pak, 2011 [120]	Human	Hip osteonecrosis	AMSCs + PRP + HA	Autologous subcutaneous abdominal fat	MRI improvements reflected in pain and functional recovery
Pak, 2012 [121]	Human	AVN of the femoral head	AMSCs + PRP + HA	Autologous subcutaneous abdominal fat	Complete bone regeneration at 16 months follow-up with improved symptoms
Pak et al., 2014 [118]	Human	AVN of the femoral head	AMSCs + PRP	Autologous subcutaneous abdominal fat	Complete MRI resolution at 18 months follow-up with improved VAS and ROM
Lee et al., 2015 [110]	Race horses	Long bone fracture	IA injection of AMSCs	Autologous subcutaneous fat from the tail	Lower levels of proinflamatory factors in synovial fluid
Saxer et al., 2016 [109]	Human	Long bone fracure	Adipose SVF + fibrin gel + open reduction	Autologous subcutaneous abdominal fat	Formation of bone ossicles at 12 months follow-up

AMSCS: Adipose-derived Mesenchymal Stem Cells, AVN: Avascular necrosis, CSD: critical size defects, HA: hyaluronic acid, IA: intra-articular, PRP: platelet- rich plasma, ROM: Range of movement, SVF: Stromal Vascular Fraction, VAS: Visual Analogue Scale.

**Table 4:** Clinical application of AMSCs in chondral injuries.

Authors	Patients	Injury	Treatment	AMSCs Origin	Outcomes
Pak, 2011 [120]	Human	Knee OA	IA injection of adipose SVF	Autologous subcutaneous abdominal fat	MRI evidence of cartilage regeneration with VAS, FRI and ROM improvements
Pak, 2013 [154]	Human	Meniscal tear	AMSCs + PRP + HA	Autologous subcutaneous abdominal fat	MRI evidence of cartilage regeneration with VAS and FRI improvements
Hyunchul, 2014 [145]	Human	Knee OA	IA injection of AMSCs	Autologous subcutaneous abdominal fat	WOMAC and VAS scores improvemements. MRI evidenced that the size of the defect decreased while the volume of cartilage increased. Second-look arthroscopy showed regenerated cartilage
Cuervo et al., 2014 [136]	Dogs	Hip OA	IA injection of AMSCs	Autologous subcutaneous inguinal fat	ROM, VAS, functional limitation, and quality of life improvements
Vilar et al., 2014 [143]	Dogs	Hip OA	IA injection of AMSCs	Autologous subcutaneous inguinal fat	PVF and VI improvements aftr the treatment
Pak, 2014 [156]	Human	Meniscal tear	AMSCS + PRP + HA	Autologous subcutaneous abdominal fat	Almost complete disappearance of the torn meniscus at 3 months after treatment
Pers et al., 2016 [147]	Human	Knee OA	IA injection of AMSCs	Autologous subcutaneous abdominal fat	Improvements in pain levels and function after 6 months follow-up
Hyunchul, 2017 [146]	Human	Knee OA	IA injection of AMSCs	Autologous subcutaneous abdominal fat	Clinical, functional, and MRI improvements after a 2-year follow-up
Freitag et al., 2017 [151]	Human	Post-traumatic isolated chondral defect of the patella	IA injection of AMSCs	Autologous subcutaneous abdominal fat	Complete fill of the chondral defect with normal hyaline-like cartilage. Improvements in pain and functional scales
Freitag et al., 2017 [152]	Human	Osteochondritis disecans	IA injection of AMSCs	Autologous subcutaneous abdominal fat	Improvements in cartilage volume with normal hyaline-like cartilage regeneration
Spasovski et al., 2018 [26]	Human	Knee OA	IA injection of AMSCs	Autologous subcutaneous abdominal fat	MRI evidenced cartilage restoration with clinical improvements within 6 months that persisted during at least 18 months
Song et al., 2018 [148]	Human	Knee OA	Three IA injections of AMSCs within 48 h	Autologous subcutaneous abdominal fat	Improved pain, function, and cartilage volume at 24 months follow-up
Panni et al., 2019 [149]	Human	Knee OA	IA injection of AMSCs + debridment arthroscopy	Autologous subcutaneous abdominal fat	Clinical and functional scores improvements at mid-term follow up, especially patients with higher pre-operative VAS score
Olsen et al., 2019 [144]	Dogs	Elbow OA	3 intravenous injection of AMSCs	Autologous subcutaneous inguinal fat	Improved activity and behavior reported by owners. No changes in synovial fluid biomarkers and mean peak vertical force

AMSCs: Adipose-derived Mesenchymal stem cells, FRI: Functional Rating Index, HA: Hyaluronic acid, IA: Intra-articular, MRI: magnetic resonance imagin, OA: Osteoarthritis, PRP: Platelet-rich plasma, PVF: Peak vertical force, ROM: Range of movement, SVF: Stromal vascular fraction, VAS: Visual Analogue Scale, VI: Verical impulse, WOMAC: Western Ontario and MCMaster Universities Osteoarthritis Index.

## Abbreviations

AMSCs	Adipose-derived mesenchymal stem cells
AVN	Avascular necrosis
BM-MSCs	Bone marrow mesenchymal stem cells
CSD	Critical size defect
DMD	Duchenne muscular dystrophy
DO	Distraction osteogenesis
FGF	Fibroblast growth factor
FRI	Functional rating index
GDF 5	Growth differentiation factor 5
GF	Growth factors
HA	Hyaluronic acid
HGF	Hepatocyte growth factor
IA	Intra-articular
IGF	Insulin growth factor
IL	Interleukin
ISCT	International Society for Cell Therapy
KOOS	Knee Injury and Osteoarthritis Outcome Score
MRI	Magnetic resonance imaging
MSCs	Mesenchymal stem cells
MSK	Musculoskeletal
NPRS	Numeric Pain Rating Scale
NSAIDs	Non-steroideal anti-inflammatory drugs
OA	Osteoarthritis
PRGF	Plasma Rich in Growth Factors
PRP	Platelet rich plasma
PVF	Peak vertical force
RM	Regenerative medicine
ROM	Range of movement
SDF-1	Stromal cell derived factor-1
SFDLT	Superficial flexor digitorium longus tendon
SVF	Stromal vascular fraction
TGF-β	Transformig growth factor β
TNF-α	Tumor necrosis factor α
VAS	Visual analogue scale
VEGF	Vascular endothelial growth factor
VI	Vertical impulse
WOMAC	Western Ontario and MCMaster Universities Osteoarthritis Index
